# Increased circulating chemerin in patients with advanced carotid stenosis

**DOI:** 10.1186/s12872-018-0803-7

**Published:** 2018-04-13

**Authors:** Adrian Kammerer, Holger Staab, Maria Herberg, Christine Kerner, Nora Klöting, Gabriela Aust

**Affiliations:** 1 Research Laboratories; Clinic for Visceral, Transplantation, Thoracic and Vascular Surgery, Leipzig University, Liebigstr. 19, D-04103 Leipzig, Germany; 2 Clinic for Visceral, Transplantation, Thoracic and Vascular Surgery, University Medical Centre Leipzig, Leipzig, Germany; 3 Interdisciplinary Centre for Bioinformatics, Leipzig University, Leipzig, Germany; 4 IFB Adiposity Disease, Junior Research Group 2, Leipzig University, Leipzig, Germany

**Keywords:** Chemerin, Carotid stenosis, Coronary artery disease

## Abstract

**Background:**

Chemerin is an adipokine which plays a crucial role in atherosclerosis. Here, we examined whether circulating chemerin is enhanced in patients with advanced carotid stenosis.

**Methods:**

Chemerin was quantified in 178 patients prior to carotid end arterectomy (CEA) and in age- and gender-matched controls (*n* = 163). Chemerin levels were related to anthropometric, clinical and metabolic characteristics of the patients.

**Results:**

Chemerin levels were higher in patients compared to controls (*p* <  0.001). Chemerin correlated to parameters associated with inflammation such as C-reactive protein (CRP, p <  0.001), leukocyte blood count (p <  0.001) and circulating TNF-α (*p* = 0.004) in the patients. Chemerin levels did not differ between asymptomatic (*n* = 93) and symptomatic patients who experienced an ischemic event within 6 months prior to CEA (*n* = 85). However, in the case of high-grade carotid stenosis (≥ 90%), chemerin levels were higher in symptomatic (*n* = 44) compared to asymptomatic patients (*n* = 41, *p* = 0.014). Chemerin was increased in patients with (*n* = 50) compared to patients without (*n* = 128) coronary artery disease (CAD, *p* = 0.002). A high level of chemerin increases the risk for CAD in patients (*p* = 0.0013).

**Conclusions:**

Circulating chemerin is increased and correlates to inflammatory parameters in patients with advanced carotid stenosis.

## Background

Cardio- and cerebrovascular diseases are the primary cause for mortality worldwide with 17.3 million deaths per year [1]. Stroke is the leading cause of acquired disability in adults and the second leading cause of death in patients aged > 60 years [2]. 87% of all strokes are ischemic due to the lack of blood flow, and 20% of all ischemic strokes result from a stenosis of extracranial vessels that nourish the brain such as the internal carotid artery [3, 4]. In most cases, the underlying cause of cardio- and cerebrovascular events is atherosclerosis, a chronic inflammatory disease leading to arterial luminal narrowing. In Germany up to one million elderly people have a stenosis grade of the carotid artery > 50% [5]. Major risk factors for atherosclerosis are adiposity, hypertension, diabetes, tobacco abuse and dysregulated blood lipids.

Adipose tissue secretes hormones and cytokines that regulate local and systemic metabolic processes and inflammatory responses. Chemerin is an adipokine first described to be located in inflammatory cells [6] and to chemoattract antigen-presenting cells as dendritic cells and macrophages [6]. Chemerin is the only known ligand for the G protein-coupled receptor chemokine-like receptor 1 (CMKLR1) and the G protein-coupled receptor-1 (GRK1), and also binds to the chemokine receptor-like 2 (CCRL2). The majority of biological functions for chemerin have been attributed to activation of CMKLR1 [7]. Both, chemerin and CMKLR1 are present at high levels in adipose tissue regulating adipogenesis and adipocyte metabolism [8]. Moreover, CMKLR1 is expressed in the tunica media and endothelial layer of arteries, where chemerin contributes to vasoconstriction [9]. Chemerin is increased in the inflamed endothelial tissue where it exerts its chemotactic action and attracts further immune cells [10, 11], and in foam and vascular smooth muscle cells of atherosclerotic lesions [3]. The data suggest a regulatory role of this adipokine in atherosclerosis.

Here, we examined whether chemerin contributes to atherosclerosis and the underlying inflammatory response. We quantified circulating chemerin in patients with advanced carotid stenosis prior to carotid endarterectomy (CEA) in age- and gender-matched controls and related chemerin levels to anthropometric, clinical and metabolic characteristics of the patients.

## Methods

### Patients and controls

In the first cohort, a total of 178 consecutive Caucasian men (*n* = 121) and women (*n* = 57) with extracranial carotid artery stenosis were included in the study. Patients, who were submitted for carotid endarterectomy (CEA) to the Clinic for Visceral, Transplant, Thoracic and Vascular Surgery (VTTG), Leipzig University, were recruited without any inclusion or exclusion criteria. According to the current valid guidelines of the European Society of Vascular Surgery (ESVS) [12] and the American Heart Association (AHA) [13], we graduated the luminal narrowing according to the NASCET criteria [14]. Two experienced vascular radiologists carried out assessments in consensus using axial source images as well as curved planar reformations and digital subtraction angiography.

Patients were classified into two groups, symptomatic (*n* = 85) or asymptomatic (*n* = 93), based on whether a patient had experienced an ischemic event (ipsilateral stroke, transient ischemic attack, or amaurosis fugax) within 6 months prior to CEA or not. Asymptomatic patients with carotid stenosis were identified randomly during clinical examination regarding coronary artery disease (CAD), peripheral artery disease, or an ischemic event in past medical history, that is > 6 months from the date of admission to hospital. Furthermore, patients with carotid stenosis were classified into patients with CAD (*n* = 50) and no CAD (*n* = 128). Coronary angiography was performed in multiple projections. CAD was diagnosed as the presence of one or more vessels with ≥50% lumen narrowing.

In the second cohort, a total number of 163 control subjects were enrolled during adiposity consultation at the Department of Medicine, Leipzig University. Clinical parameters of this cohort were assessed as described previously [15].

### Blood samples and assays

Peripheral venous blood was collected between 8 and 10 am after an overnight fast; in the case of carotid patients 1 day before CEA. Blood was immediately centrifuged at 2500 g for 20 min. Plasma samples were stored at − 80 °C and thawed only once. Measurement of routine biochemical parameters was performed with standard procedures [16, 17]. Chemerin was quantified with a sandwich ELISA (BioVendor GmbH, Kassel, Germany; sensitivity: 0.1 ng/ml). TNF-α was measured using an ELISA (R&D Systems, Inc., Minneapolis, USA; sensitivity: 0.1 ng/ml, reference range of normal TNF-α- values: < 5 ng/ml).

### Statistics

Parameters were tested against a normal distribution using the one sample Kolmogorov-Smirnov test. Non-normally distributed parameters were logarithmically transformed to approximate a normal distribution. Mean and standard error of mean (SEM), for non-normally distributed parameters median and the interquartile range [25th–75th percentiles], were used. A comparison between different groups regarding categorical variables was carried out using the Chi-square test. Two independent samples were compared using the Students’s t-test for normally distributed data and the Mann-Whitney U-test for non-normally distributed data, respectively. Correlations between two quantitative variables were assessed using the Pearson coefficient for normally distributed data and Spearman Rho for non-normally distributed data, respectively. Receiver Operating Characteristic (ROC) analysis was used to assess the diagnostic power of chemerin for discrimination between CAD and no CAD patients. The observed area under the ROC curve (AUC) was tested against the null-hypothesis (AUC = 0.5) using the Mann-Whitney U-test. A logistic regression model was performed to further evaluate whether high chemerin increases the risk for CAD. All statistical computations were performed using SPSS version 24.0 (Chicago, IL, USA). *P* values less than 0.05 were considered as significant.

## Results

### Higher circulating chemerin levels in patients with advanced carotid stenosis

Baseline information on anthropometric, clinical and metabolic characteristics of the 178 patients and 163 control subjects are shown in Table 1. Age and gender distribution did not differ between both groups, allowing for an unbiased comparison of the other parameters. The body mass index (BMI; *p* = 0.001), total cholesterol (*p* = 0.009), low density lipoprotein (LDL) cholesterol (p = 0.001), and high density lipoprotein (HDL) cholesterol (*p* = 0.005) were lower, and the leukocyte blood count (p = 0.001), CRP (*p* = 0.010) and triglycerides (*p* = 0.042) were higher in patients compared to controls. 63.5% of the patients received statins, HMG-CoA reductase inhibitors, which effectively reduce circulating LDL cholesterol (statin-untreated 3.5 [3.0–4.1] mmol/l; statin treated 2.4 [2.1–3.1] mmol/l; *p* <  0.001). HbA1c (*p* = 0.161) and fasting glucose (*p* = 0.166) did not differ between both groups.

**Table 1:** Clinical and anthropometric characteristics of carotid stenosis patients subdivided into asymptomatic/symptomatic and patients without/with coronary artery disease (CAD)

	controls	patients		patients			patients		
			*p*-value	asymptomatic	symptomatic	p-value	no CAD	CAD	p-value
number	163	178		93	85		128	50	
age	65.2 ± 0.7	66.9 ± 0.6	0.058	68.0 [62.5–73.5]	68.0 [59.0–73.0]	0.924	67.0 [59.0–71.8]	71.5 [66.0–75.2]	**0.001**
gender (m: w)	105: 58	121: 57	0.487	66: 27	55: 30	0.371	82: 46	39: 11	0.073
BMI (kg/m^2^)	29.1 [25.7–31.5]	26.6 [24.5–29.3]	**< 0.0001**	26.9 [24.8–28.9]	26.2 [23.9–30.1]	0.789	26.6 [24.2–29.0]	27.1 [25.1–30.0]	0.286
Stenosis (%)	nd	85.0 [85.0–90.0]		85.0 [80.0–90.0]	85.0 [85.0–90.0]	0.668	85.0 [85.0–90.0]	85.0 [80.0–90.0]	0.690
(Ex) smoker (%)	nd	69.9		69.7	70.2	0.934	74.4	63.3	0.229
hypertension (%)	nd	93.3		95.7	90.6	0.174	91.4	98.0	0.115
systolic blood pressure (mmHg)	nd	140.0 [130.0–160.0]		140.0 [130.0–160.0]	140.0 [130.0–156.3]	0.617	145.0 [130.0–160.0]	140.0 [121.8–150.0]	0.073
diastolic blood pressure (mmHg)	nd	80.0 [70.00–90.0]		80.0 [70.0–90.0]	80.0 [70.0–90.0]	0.570	80.0 [70.0–90.0]	80.0 [70.0–85.0]	0.461
T2DM (%)	49.1	40.4	0.111	38.7	42.4	0.621	33.6	58.0	**0.003**
fasting glucose (mmol/l)	5.6 [4.9–6.8]	5.9 [5.1–7.1]	0.166	5.7 [5.1–7.1]	5.9 [5.1–7.1]	0.709	5.7 |5.1–7.0|	6.0 [5.1–7.8]	0.482
HbA1c (%)	5.8 [5.4–6.3]	5.9 [5.5–6.5]	0.161	6.0 [5.5–6.6]	5.9 [5.5–7.1]	0.566	5.8 [5.4–6.4]	6.2 [5.8–7.8]	**0.010**
total cholesterol (mmol/l)	5.2 ± 0.1	4.9 ± 0.1	**0.009**	5.1 ± 0.1	4.6 ± 0.1	**0.006**	4.9 ± 0.1	4.7 ± 0.1	0.187
LDL cholesterol (mmol/l)	3.2 [2.8–3.8]	2.8 [2.2–3.6]	**0.001**	2.9 [2.3–3.9]	2.5 [2.0–3.3]	**0.047**	2.9 [2.2–3.8]	2.6 [2.2–3.2]	0.245
HDL cholesterol (mmol/l)	1.4 [1.1–1.7]	1.2 [1.0–1.5]	**0.005**	1.3 ± 0.05	1.2 ± 0.04	**0.006**	1.2 [1.0–1.5]	1.2 [0.9–1.5]	0.907
triglyceride (mmol/l)	1.5 [1.1–2.2]	1.7 [1.2–2.6]	**0.042**	1.7 [1.2–2.7]	1.7 [1.2–2.6]	0.791	1.8 [1.2–2.6]	1.5 [1.1–2.8]	0.691
Leukocyte blood count (exp9/l)	6.7 [5.6–8.4]	7.6 [6.1–8.9]	**0.001**	7.5 [6.0–9.0]	7.8 [6.3–8.8]	0.382	7.7 [6.3–9.3]	7.2 [5.8–8.5]	0.068
CRP (mg/l)	2.5 [1.3–5.1]	3.5 [1.6–8.0]	**0.010**	2.5 [1.4–5.6]	4.5 [1.8–9.5]	**0.027**	3.2 [1.5–8.0]	3.9 [1.6–7.9]	0.863
TNF-α (pg/ml)	nd	2.7 [2.2–4.9]		3.1 [2.5–4.4]	2.6 [2.1–3.9]	0.191	2.6 [2.1–4.7]	3.0 [2.2–3.9]	0.983
chemerin (ng/ml)	185.7 [151.8–208.2]	229.9 [200.6–282.0]	**< 0.001**	223.2 [197.9–277.7]	240.2 [201.6–286.8]	0.498	223.2 [193.3–272.2]	255.9 [207.9–327.2]	**0.002**
statine treatment (%)	nd	63.5		58.1	69.4	0.116	60.2	72.0	0.140
antithrombotic treatment (%)	nd	88.8		89.2	88.2	0.831	89.8	86.0	0.466

Mean ± SEM, for non-normally distributed parameters median [interquartile range] are given; significant differences are in bold

*BMI* body mass index, *CAD* coronary artery disease, *CRP* C-reactive protein, *HDL* high density lipoprotein, *LDL* low density lipoprotein, T2*DM* type 2 diabetes mellitus, *TNF*-α tumor-necrosis factor-α

**Fig. 1:**
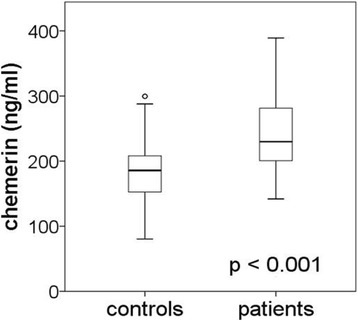
Higher circulating chemerin in patients with advanced carotid stenosis. Chemerin levels differ between control subjects (n = 163) and patients (*n* = 178; median and interquartile range, whiskers 95% percentile; t-test)

**Fig. 2:**
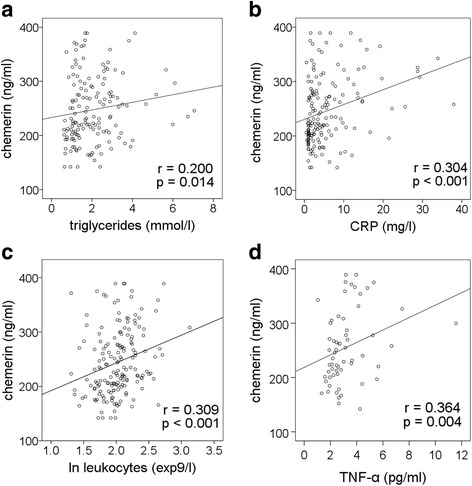
Circulating chemerin correlates to triglycerides (**a**) and parameters of inflammation (**b**: C-reactive protein, CRP; **c**: peripheral leukocyte count; **d**: tumor-necrosis factor- α, TNF-α)

Circulating chemerin levels were increased 1.3 fold in patients compared to controls (*p* <  0.001, Fig. 1). Within the patient group women had higher chemerin levels (268.89 [215.1–319.92] ng/ml) compared to men (231.42 [193.00–267.82] ng/ml; *p* <  0.0001). Correlations of the chemerin level to patient characteristics are summarized in Table 2. Chemerin levels correlated positively to triglycerides (*p* = 0.014, Fig. 2a), CRP (p <  0.001, Fig. 2b), leukocyte blood count (p <  0.001, Fig. 2c), and circulating TNF-α (*p* = 0.004, Fig. 2d). No correlations between chemerin and the various parameters were found in control subjects.

**Table 2:** Correlation of chemerin levels to anthropometric and metabolic characteristics of patients with carotid stenosis, significant differences are in bold

parameter	r	p
age	0.121	0.111
BMI	0.097	0.208
HbA1c	0.035	0.726
fasting glucose	−0.024	0.780
total cholesterol	−0.037	0.657
LDL cholesterol	−0.064	0.440
HDL cholesterol	−0.046	0.580
triglycerides	0.200	**0.014**
leukocyte blood count	0.309	**< 0.001**
CRP	0.304	**< 0.001**
TNF-α	0.360	**0.004**

### Chemerin levels are higher in patients with CAD compared to CAD-unaffected patients

Chemerin levels did not differ between asymptomatic (*n* = 93) and symptomatic patients (*n* = 85, Tab. 1). We further examined patients with high grade stenosis (≥ 90.0% luminal narrowing, n = 85). Symptomatic patients (*n* = 44) showed higher chemerin levels compared to asymptomatic patients (*n* = 41, *p* = 0.014) with high grade stenosis.

**Fig. 3:**
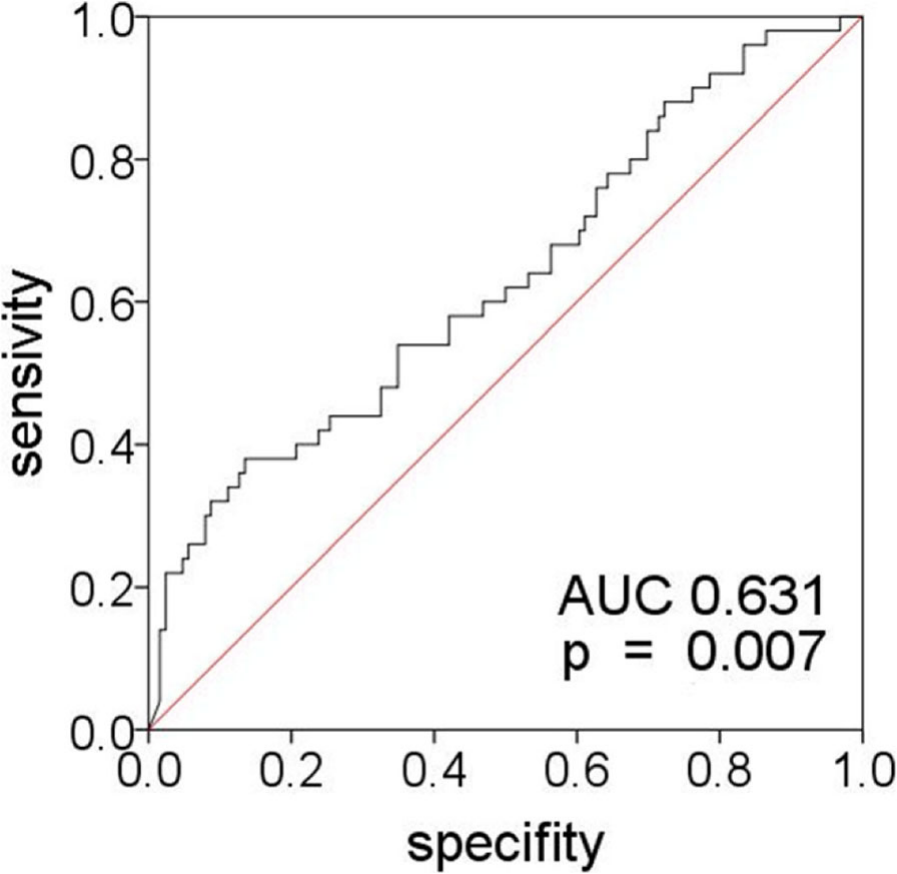
AUC analysis of chemerin levels to predict coronary artery disease (CAD)

50 out of 178 (50/178) patients were diagnosed for CAD. Patients with CAD had higher chemerin levels compared to CAD-unaffected patients (*n* = 128, *p* = 0.002, Tab. 1). Construction of a ROC curve indicates that chemerin can determine whether patients had CAD or not (AUC 0.631 [0.535–0.724], *p* = 0.007, Fig. 3). To further examine this hypothesis, we performed a logistic regression. Thereby we found that chemerin significantly increases the risk for CAD (*p = 0.0013)*. Exactly one unit increase in chemerin increases the odds of having CAD by 0.2% (odds ratio = 1.002 [1.001–1.003]).

## Discussion

In the present study, we found higher circulating chemerin levels in patients with advanced carotid stenosis compared to age- and gender-matched controls.

Atherosclerosis is considered as chronic inflammation of the arteries [18]. Consistently, parameters of inflammation such as the CRP and leukocyte blood count were higher in our patients compared to controls. Both, CRP and the leukocyte blood count as well as circulating TNF-α correlated to chemerin in our patients confirming several studies in which chemerin and inflammatory parameters were strongly associated [19–23]. Chemerin directly affects the pathomechanism of atherosclerosis. Circulating chemerin increased after carotid balloon injury in mice, a model to induce atherosclerosis [11]. Chemerin siRNA knockdown inhibited the proliferation of vascular smooth muscle cells induced by platelet-derived growth factor-BB and pro-inflammatory chemokines in vitro and prohibited carotid neointimal hyperplasia and pro-inflammatory chemokines in vivo after angioplasty [11]. Moreover, high levels of chemerin were associated with plaque instability, scored in histological sections [24]. This is likely due to the chemotactic effect of this adipokine on plaque macrophages, which changes the strength of the plaques. However, circulating chemerin was not associated with the cerebrovascular symptomatology of the patients [24], which is also the case in our study. Because the degree of carotid artery stenosis is an established risk factor for an ischemic stroke [25], we re-evaluated patients with a high-grade stenosis concerning cerebrovascular symptomatology. Symptomatic patients had higher chemerin levels than asymptomatic patients with ≥90% luminal narrowing.

Our study confirmed the association of chemerin to CAD. Chemerin levels were elevated in metabolic syndrome patients with CAD [26] and positively related to the Gensini score, which evaluates the circulation in coronary arteries, in Chinese adults [27]. In a further study chemerin mRNA expression in epicardial adipose tissue was associated with coronary atherosclerosis [28] but in this study circulating chemerin and the Gensini score were not associated. Epicardial and periadventitial adipose tissue are not only innocent bystanders, but play a role in vascular pathophysiology through adipokine secretion. Indeed, periadventitial fat and foam cell chemerin immunopositivity correlated to the severity of atherosclerosis in human aortic and coronary artery samples [3]. In part, an autocrine positive feedback loop is established: both the adipokine and its receptor CMKLR1 are expressed in vascular smooth muscle and foam cells in atherosclerotic lesions of aortic and coronary vessels [3]. Interestingly, serum chemerin was found to be associated with the brachial ankle pulse wave velocity (baPWV), a parameter of arterial stiffness [22].

Adipose tissue is one of the major sources of chemerin [23]. Consistently, in several studies serum chemerin correlated to the BMI and waist circumference [21–23, 29]. In our study chemerin and the BMI were not associated. The BMI was even lower in patients compared to controls suggesting other cellular sources of chemerin such as the abdominal adipose tissue in patients with advanced carotid stenosis. Chemerin is widely distributed with particularly high levels in the liver [8] and, as discussed above, it is expressed in the affected vascular smooth muscle and foam cells of atherosclerotic lesions and fat enveloping arteries. Circulating, activated immune cells or thrombocytes, excreting chemerin during their activation [30], may be other cellular sources.

## Conclusions

Circulating chemerin is elevated in patients with advanced carotid stenosis and CAD. It is related to parameters of systemic inflammation and in high grade stenosis to cerebrovascular symptomatology.

## Abbreviations

AUC: Area under the ROC curve
BMI: Body mass index
CAD: Coronary artery disease
CEA: Carotid end arterectomy
CMKLR1: Chemokine-like receptor 1
CRP: C-reactive protein
HDL: High density lipoprotein
LDL: Low density lipoprotein
ROC: Receiver Operating Characteristic
T2DM: Type 2 diabetes mellitus
TNF: Tumor-necrosis factor
